# Clonal architectures predict clinical outcome in clear cell renal cell carcinoma

**DOI:** 10.1038/s41467-019-09241-7

**Published:** 2019-03-18

**Authors:** Yi Huang, Jiayin Wang, Peilin Jia, Xiangchun Li, Guangsheng Pei, Changxi Wang, Xiaodong Fang, Zhongming Zhao, Zhiming Cai, Xin Yi, Song Wu, Baifeng Zhang

**Affiliations:** 1 Department of Computer Science and Technology, School of Electronic and Information Engineering, Xi’an Jiaotong University, Xi’an, Shaanxi 710049 China; 2 Geneplus-Beijing, Beijing, 102206 China; 3 Center for Precision Health, School of Biomedical Informatics, The University of Texas Health Science Center at Houston, Houston, TX 77030 USA; 4 Department of Epidemiology and Biostatistics, Tianjin Medical University Cancer Institute and Hospital, Tianjin, 300060 China; 5 The Second Affiliated Hospital of Guangzhou University of Chinese Medicine, Guangzhou, 510120 Guangdong China; 6 The Third Affiliated Hospital of Shenzhen University, Shenzhen, 518001 Guangdong China

## Abstract

The genetic landscape of clear cell renal cell carcinoma (ccRCC) had been investigated extensively but its evolution patterns remained unclear. Here we analyze the clonal architectures of 473 patients from three different populations. We find that the mutational signatures vary substantially across different populations and evolution stages. The evolution patterns of ccRCC have great inter-patient heterogeneities, with del(3p) being regarded as the common earliest event followed by three early departure points: *VHL* and *PBRM1* mutations, del(14q) and other somatic copy number alterations (SCNAs) including amp(7), del(1p) and del(6q). We identify three prognostic subtypes of ccRCC with distinct clonal architectures and immune infiltrates: long-lived patients, enriched with *VHL* but depleted of *BAP1* mutations, have high levels of Th17 and CD8^+^ T cells while short-lived patients with high burden of SCNAs have high levels of Tregs and Th2 cells, highlighting the importance of evaluating evolution patterns in the clinical management of ccRCC.

## Abstract (web summary)

Clear cell renal cell carcinoma (ccRCC) is a urogenital cancer with a well-defined genetic landscape. Here, the authors analyse the clonal architecture of ccRCC patients from three populations, and find prognostic subtypes linked to immune infiltrates and clonal architecture.

## Introduction

Clear cell renal cell carcinoma (ccRCC) is one of the most lethal forms of urogenital tumors and over 140,000 cases are estimated to die of ccRCC annually all over the world^1,2^. To understand the etiology of ccRCC, genetic alterations in ccRCC had been screened in large cohorts of patients previously^3–8^. These large-scale studies revealed that the genetic landscape of ccRCC is characterized by the high prevalence of somatic copy number alterations (SCNAs) and a relatively low burden of somatic substitutions^3–8^. Arm-level SCNAs including del(3p), amp(5q), amp(7q), del(9p), amp(12p) and del(14q) were found to affect 45–85% of ccRCC patients and several driver genes including *VHL*, *PBRM1*, *SETD2* and *BAP1* were observed to be mutated in 10–50% of patients. Integrative analysis of the genetic and clinical information demonstrated that certain SCNAs or mutated driver genes could be potential prognostic markers^9^. For instance, *BAP1* mutations, mutually exclusive with *PBRM1* mutations, can predict poor clinical outcome in ccRCCs independently^10–12^. The development of human cancers is driven by the stepwise accumulation of somatic alterations and mutations acquired at different stages of tumor evolution are likely to be associated with different clinical outcomes^13^. However, the temporal order of acquiring the somatic events during ccRCC evolution as well as their potential clinical effects had not been fully studied.

Accumulating evidence suggested that ccRCCs had startling intratumor heterogeneity (ITH) which may have great influence on tumor metastasis and therapeutic responses^14,15^. Multi-region exome sequencing of several ccRCC patients showed that different sections of the same tumor masses harbored somatic events co-existing in distinct subclones which evolved following a branched pattern^14,15^. These studies found that del(3p) and inactivation of *VHL* were trunk events while most of the other driver aberrations were subclonal. However, the numbers of patients analyzed in these studies were quite small. It is necessary to evaluate the inter-patient differences in ITH and evolution patterns systematically and to further analyze their influence on clinical outcomes in large cohorts of ccRCCs. Previous studies had demonstrated the possibility of reconstructing the clonal architecture of single tumor biopsy by estimating the fraction of tumor cells carrying either SCNAs or single-nucleotide variants (SNVs)^16–19^. Nevertheless, no previous study had quantified the cancer cell fractions (CCFs) of both SCNAs and SNVs simultaneously within the same ccRCCs and thus had some limitations for reconstructing the evolution history of ccRCCs.

To more fully characterize the clonal diversities of ccRCCs, we obtain the published large-scale genomic data from The Cancer Genome Atlas (TCGA) and the Japanese population^3,4^. Additionally, we also sequence the whole-genomes of a cohort of Chinese ccRCC patients. We infer the temporal order of the somatic events frequently occurred in ccRCCs, compare the mutational signatures and evolution patterns among different populations and evaluate their clinical relevance in a total of 473 ccRCC patients. Our results generate a full picture of variations in mutational signatures and ITH during ccRCC evolution, propose putative evolution models of ccRCC development and discover several clonal or subclonal events as potential prognosis markers. We further perform molecular subtyping of ccRCC based on the CCFs of all potential prognostic events and characterize the expression and immune features of the different prognostic genomic subtypes.

## Results

### Mutational signature analysis of ccRCC

Of the ccRCC-473 cohort, 328 were TCGA samples, 104 were Japanese and 41 were Chinese. We identified the somatic SNVs from the Japanese and Chinese samples using MuTect2 and generated the profiles of SCNAs with the whole-exome or whole-genome sequencing data using ReCapSeg^20,21^. After several preprocessing and filtering steps, a total of 40,697 somatic SNVs and 9,451 SCNA segments were kept for downstream analysis in the ccRCC-473 cohort. We estimated the CCF of each SNV and SCNA in all samples^16,17,22^. A somatic event was defined as clonal if the CCF harboring the SNV or SCNA was ≥0.95 with probability >0.5 and subclonal otherwise^16,17,22^. According to this criterion, we identified 12,458 and 4,143 subclonal SNVs and SCNA segments, respectively. Of the coding SNVs, 68.1% were clonal mutations.

**Fig. 1:**
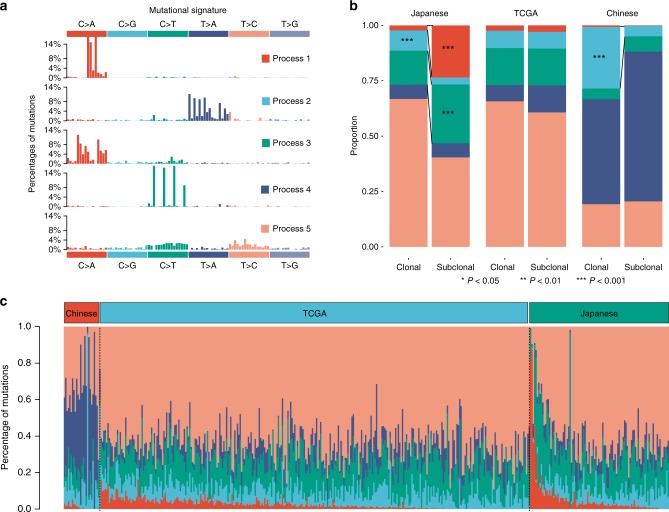
The distribution of mutational signatures across different cohorts. **a** Five distinct mutational signatures identified by NMF analysis of the matrix of mutation proportion across tumors from different populations. **b** Comparison of mutational signatures between clonal and subclonal mutations. Enrichment of mutational signatures between clonal and subclonal mutations was determined by Fisher test of the relative contribution of each signature in all patients. **c** Mutational exposures (number of mutations) attributed to each mutation signature in each patient

The mutational signatures can reflect the potential influence of previous exposures to different carcinogens as well as the associated DNA damage and repair processes operating in ccRCC tumors. We performed mutational signature analysis by stratifying the SNVs according to their trinucleotide mutational contexts^23–25^. Of the five independent mutational signatures we identified (Fig. 1a), three matched known signatures (cosine similarities ranged from 0.84 to 0.93) that had been described in the Catalogue of Somatic Mutations in Cancer (COSMIC) database (Supplementary Fig. 1 and Supplementary Data 1)^23–25^. The signature matched COSMIC signature 1 (denoted as process 4 in Fig. 1a), characterized by C>T transitions at CpG dinucleotides, was observed in different tumor types and was likely to result from 5-methlcytosine deamination^23–25^. The signature closely resembling COSMIC signature 22 (process 2 in Fig. 1a), characterized by T>A transversions at CT [A/G] (where the mutated T is preceded by C and followed by A or G), was found in urothelial carcinomas with known exposures to aristolochic acid^23–25^. The signature closely resembling COSMIC signature 5 (process 5 in Fig. 1a), characterized by a broad spectrum of base changes, was also present in different tumor types and was suggested to be associated with *ERCC2* mutations in bladder cancer^26^. Two other signatures, characterized by C>A transversions at GC [A/T] and [C/T] C [A/T] motifs, did not match any known COSMIC signatures well (with maximum cosine similarities of 0.59 and 0.79 to signatures 29 and 4, respectively) and maybe occur in ccRCC only (processes 1 and 3 in Fig. 1a).

To investigate the heterogeneity in mutational signatures, we compared the activity of each mutational signature among three patient cohorts. Overall, process 5 was active in all three ccRCC cohorts while the activities of four other signatures varied substantially among different cohorts (Fig. 1b, c). Process 1 was relatively enriched in Japanese patients while process 4 was prevalent in Chinese patients, demonstrating the presence of diversity in ccRCC mutation signatures among populations (Fig. 1b, c). To further explore whether mutational signatures varied during ccRCC evolution, we analyzed the distribution of clonal and subclonal SNVs for each signature among different populations. Among the five mutational processes, process 2 was significantly more prevalent in clonal than subclonal mutations in both Japanese-104 cohort and Chinese-41 cohort (*P* < 0.0001 and *P* < 0.0001, respectively). In contrast, processes 1 and 3 were significantly enriched with subclonal mutations in Japanese-104 cohort (*P* < 0.0001 and *P* < 0.0001), suggesting processes 1 and 3 as mutational processes contributing to the accumulation of subclonal mutations in Japanese patients (Fig. 1b).

### The clonal architectures of ccRCC

**Fig. 2:**
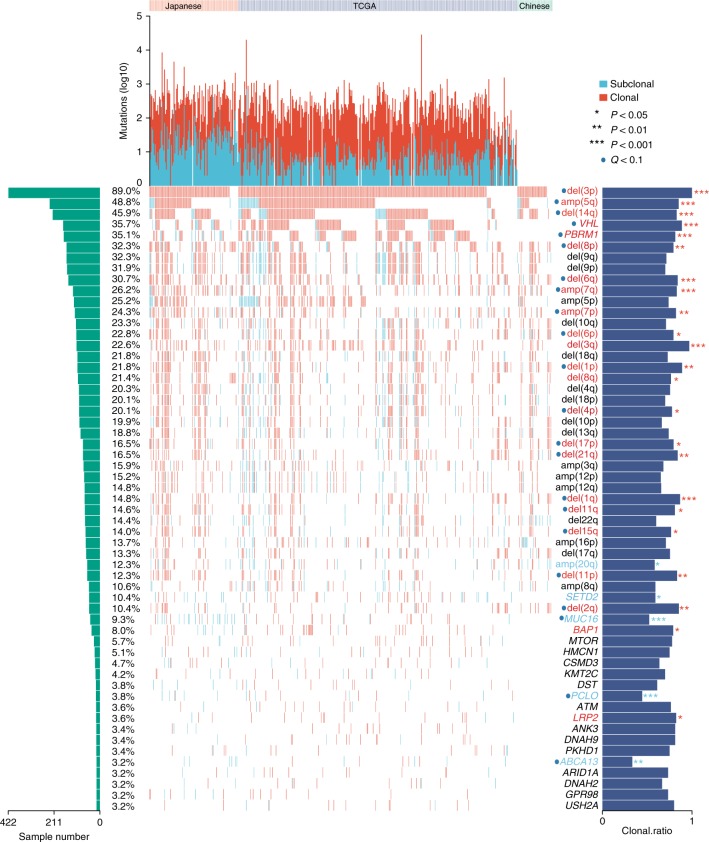
The clonality of frequently altered genes and arm-level SCNAs in ccRCC. The top panel shows the prevalence of clonal (red) and subclonal (blue) SNVs in the Japanese and TCGA ccRCC cohorts. The bottom panel shows the clonal or subclonal state of each somatic event (row) in all patients. The names of genes and SCNAs significantly enriched with clonal or subclonal alterations are labeled in red and blue, respectively

To explore the contribution of SCNAs during ccRCC evolution, we estimated the CCF of each SCNA segment in each sample and calculated the fraction of samples harboring clonal or subclonal SCNAs at the chromosome arm level. The fractions of samples harboring clonal or subclonal events were generally similar among the three cohorts for almost all the arm-level SCNAs except for del(16p) and del(17p), both of which showed an elevated level of subclonal event in the Eastern Asian populations (Supplementary Fig. 2). We identified 36 frequent arm-level SCNAs that were altered in at least 10% of patients in the ccRCC-473 cohort. About 53% (19/36) and 3% (1/36) of the frequent arm-level SCNAs showed significant enrichment of clonal and subclonal events in the ccRCC-473 cohort, respectively (Fig. 2). Consistent with previous studies in other cancers^13^, multiple arm-level driver SCNAs in ccRCC often appeared as clonal events in the majority of patients (Fig. 2), including del(3p), amp(5q), amp(7) and del(14q). These data suggested that most of the arm-level driver SCNAs were shared by different patient cohorts and were likely clonal events which occurred early during ccRCC evolution.

We identified 21 genes that were mutated in at least 3% of samples in the TCGA and Japanese samples (ccRCC-432 cohort) which were analyzed by high-depth whole-exome sequencing (Fig. 2). Of these genes, three were known driver genes *VHL* (*P* < 0.0001, FDR < 0.0001), *PBRM1* (*P* *=* 0.0002, FDR = 0.001) and *BAP1* (*P* *=* 0.047, FDR = 0.1) significantly enriched with clonal non-silent mutations. These findings were generally consistent with previous multi-region sequencing of 10 ccRCCs showing that mutations in *VHL* and *PBRM1* tended to be trunk events^14^. In addition, several frequently mutated genes without well-established roles in ccRCCs were also found to be enriched with either clonal or subclonal mutations. For instance, mutations in *LRP2* (*P* *=* 0.037, FDR = 0.1) showed a tendency to be clonal while mutations in *MUC16* (*P* < 0.0001, FDR < 0.0001), *PCLO* (*P* *=* 0.0003, FDR = 0.009) and *ABCA13* (*P* *=* 0.001, FDR = 0.02) tended to be subclonal, highlighting their potential roles in either the genesis or progression of ccRCC.

### Temporal order of somatic mutation acquisitions in ccRCC

**Fig. 3:**
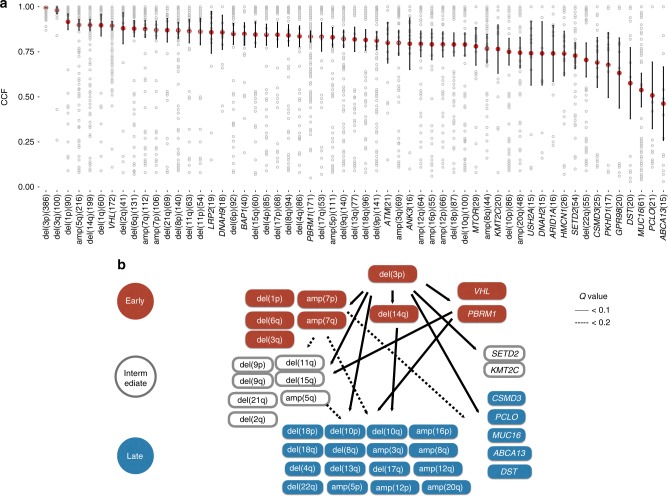
The temporal order of mutation acquisitions during ccRCC evolution. **a** The distributions of cancer cell fraction (CCF) values for the frequent somatic events. The median CCF value is shown for each gene or SCNA (red dots represent the medians and bars represent 95% confident intervals). **b** The temporal maps of mutation acquisitions in ccRCC. Temporally direct edges are drawn when two drivers are found in the same sample, one in clonal and the other in subclonal. Only driver pairs with at least five connecting edges were tested for statistical significance

To examine the probable temporal order of driver acquisitions during ccRCC evolution, we ranked the 21 frequently mutated genes and 36 arm-level SCNAs according to the distributions of CCFs in the ccRCC-432 cohort (Fig. 3a). Overall, the arm-level SCNAs had a significantly higher median CCF than all the frequently mutated genes (*P* < 0.0001). The median CCF of del(3p) was the highest among all the somatic events and several other arm-level SCNAs including del(1), amp(5q) and del(14q) also had slightly higher medians of CCFs than the well-known renal cancer driver *VHL* mutations, suggesting that the acquirement of certain arm-level SCNAs may play initialing roles in the early stage of ccRCC evolution.

We inferred the potential temporal relationship between pairs of frequent somatic events by identifying samples in which one somatic event was clonal and the other was subclonal. The clonal mutation was considered to be acquired earlier than the subclonal mutation in the same patient and a temporal ‘edge’ would be drawn from the former to the latter. We classified each frequently mutated gene or arm-level SCNA into early, intermediary or late event based on the degree of enrichment of out-going edges compared to in-going edges (Supplementary Data 2)^27,28^. We further constructed the evolutionary trajectories of ccRCC according to the temporal relationship between two somatic events in each of the 749 pairs connected by at least 5 edges (Supplementary Data 2 and Fig. 3b).

Of the different potential routes of ccRCC evolution (Fig. 3b), the earliest event del(3p) was followed by three groups of departure points: (i) somatic mutations involving *VHL* and *PBRM1*, (ii) del(14q), and (iii) arm-level SCNAs including amp(7), del(1p) and del(6q). These divergent routes of tumor evolution finally converged toward the late group of SCNA events such as deletions of chromosomes 10, 18, and 17q and amplifications of 12, 16p, and others (Fig. 3b), indicating the presence of different genomic subtypes of ccRCC with distinct evolution patterns. The existence of heterogeneity in the clonal architectures necessitated the evaluation of evolution patterns in prognosis analysis for ccRCC.

### The prognostic value of subclonal events in ccRCC

To evaluate the prognostic values of the frequently mutated genes or SCNAs, we performed Kaplan–Meier analysis of the somatic alterations and identified 18 somatic events (including del(9), amp(12), del(14q), del(1p), del(4), del(13q), amp(3q), del(11q), del(22q), del(15q), del(2q), and mutations in genes *VHL* and *BAP1*) as potential factors relevant to the prognosis of ccRCC (Supplementary Data 3). Of these genomic alterations, only mutations of *VHL* indicated good clinical outcome. We explored whether clonal or subclonal alterations would have different impact on clinical outcomes and further performed multivariate Cox regression analysis based on four covariates including age, gender, TNM stage, and Fuhrman grade.

Both clonal and subclonal alterations involving chromosomes 14q, 15q, and *BAP1* gene were associated with shortened survival time in ccRCC patients (Supplementary Data 3). However, only subclonal del(14q) and del(15q) showed significant associations with poor clinical outcomes in multivariate analysis. Under the univariate analysis model, patients with clonal del(9), del(13q), del(22q), and *HMCN1* mutations or with subclonal amp(12), del(1p), del(4q), and amp(3q) were significantly associated with shortened survival. In addition, subclonal mutations in *USH2A*, a new target shown to be mutated frequently in relapsed leukemia, were significantly associated with dismal clinical outcome in univariate analysis^29^. Under the multivariate model, only clonal *HMCN1* mutations and subclonal amp(12) were significantly associated with dismal clinical outcome (Supplementary Fig. 3 and Supplementary Data 3).

Interestingly, several subclonal arm-level SCNAs positively correlated with the TNM stages and Fuhrman grades of tumors. Comparing with tumors staged as T1 or T2, subclonal del(9), del(4), del(15q), del(1p) and amp(3q) tended to occur more frequently in tumors staged as T3 or T4 (all *P* < 0.05). High grade tumors (G3 or G4) were more likely to harbor subclonal amp(12) than the low-grade tumors (*P* < 0.0001, Supplementary Fig. 4). The above observations indicated that the evolutionary stages of mutation acquisitions had great influence on their prognostic values. Future genomic studies of ccRCC should pay more attention to the subclonal events which were generally acquired late during evolution and thus may contribute to the progression of ccRCC.

### Genomic subtyping of ccRCC based on clonal architectures

**Fig. 4:**
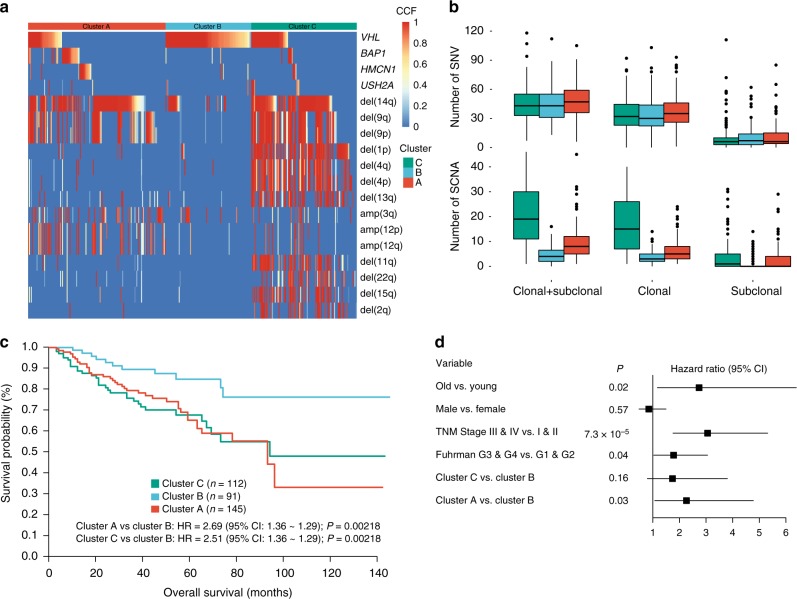
Prognostic significance of molecular subtyping in ccRCC. **a** Molecular subtyping of ccRCC based on the CCF values of 18 frequent somatic events showing associations with clinical outcomes in univariate analyses. **b** Comparison of the overall burdens of SNVs and arm-level SCNAs among different genomic subtypes of ccRCC. The *P* values are determined by Student’s *t* tests. **c** Kaplan–Meier survival curves displaying survival outcomes of different clusters. **d** The results of multivariate Cox regression analyses adjusting for age, sex, TNM staging, Fuhrman grade, and molecular subtypes. Hazards ratio (HR), 95% confidence interval (CI), and *P*-values are displayed

We next tried to identify whether there were any molecular subtypes of ccRCCs whose clonal architectures had great influence on their clinical outcomes. We performed non-negative matrix factorization (NMF) analysis of the CCFs of the eighteen frequently mutated genes and SCNAs identified to be associated with survival by the univariate analysis. In total, we identified three molecular subgroups (termed as clusters A, B, and C) whose clinical outcomes were divergent (Fig. 4a). The overall burdens of` somatic mutations were generally similar among the three clusters of patients, but patients in clusters A and C had a greater burden of arm-level SCNAs (*P* < 0.0001 and *P* < 0.0001, respectively; Fig. 4b) and had poorer probability of survival than cluster B patients (Fig. 4c) (HR = 2.69; *P* = 0.002; 95% CI: 1.36–1.29). Multivariate analysis further proved that we could predict the clinical outcome of ccRCC patients independently by clustering analysis of CCFs of the frequent somatic events (Fig. 4d).

The genomic landscapes and the potential orders of acquiring the somatic events showed great divergence among the three clusters. Almost all tumors in cluster B harbored *VHL* mutations but were devoid of *BAP1* mutations while tumors in clusters A and C were predominantly enriched with del(14q) and multiple other cluster-specific arm-level SCNAs. For instance, three SCNAs including amp(12), amp(3q) and del(9) and seven events including del(1p), del(4), del(13q), del(11q), del(22q), del(15q) and del(2q) were predominantly enriched in clusters A and C, respectively (Supplementary Data 4). A number of tumors in clusters A and C appeared to acquire del(14q) in the early stage while a number of tumors in cluster B acquired del(14q) in the late stage of evolution (Supplementary Fig. 5).

### Expression and immune features of genomic subtypes of ccRCC

**Fig. 5:**
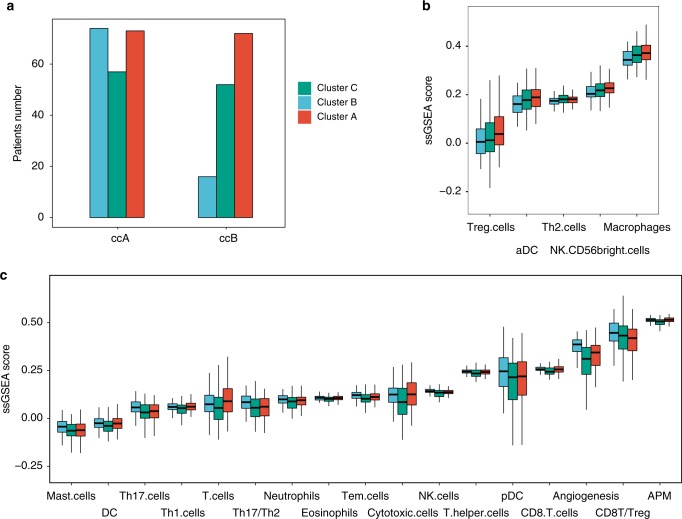
Expression and immune features of different subgroups of ccRCC. **a** The numbers of ccA and ccB tumors in each cluster are shown. **b** Signature genes downregulated in cluster B relative to clusters A and C. **c** Signature genes upregulated in cluster B relative to clusters A and C

Distinct prognostic subtypes of ccRCC (ccA and ccB) had been identified previously based on gene expression profiling^4,30^. We also subtyped our ccRCC cohorts according to their expression profiles and compared the relationship between their genomic and expression prognostic subtypes. We found that patients with the ccA expression profile, an indicator of good prognosis, were significantly enriched in cluster B (*P* < 0.0001) which also showed better clinical outcome than the other two clusters in our study (Fig. 5).

Previous evidence suggested that the tumor microenvironment of ccRCC was infiltrated with high levels of different immune components which may have different consequences on the prognosis of ccRCC^31^. We compared the relative expression levels of the immune signature genes among the three genomic prognostic clusters using ssGSEA^32^ (Fig. 5). We found that the infiltration levels of Tregs and Th2 cells were higher in clusters A and C (*P* = 0.005, FDR = 0.016 and *P* = 0.042, FDR = 0.091, respectively) than in cluster B. In contrast, the infiltration levels of Th17 and CD8^+^ T cells (*P* = 0.003, FDR = 0.011 and *P* = 0.0002, FDR = 0.002, respectively) as well as the ratios of Th17/Th2 and CD8^+^/Treg were higher in cluster B (*P* = 0.002, FDR = 0.008 and *P* = 0.001, FDR = 0.006, respectively) than clusters A and C. The Tregs and Th2 cells were often suggested to have immunosuppressive roles in promoting tumor formation and progression, whereas the cytolytic CD8^+^ T cells had been proved to be the key denominator of survival in patients with various types of solid cancers^33,34^. The infiltrating level of Th17 cells in tumor mass was negatively correlated with tumor growth/stage in some human cancers^35^. Th17 cells could elicit antitumor effects by inhibiting Tregs, inducing the expression of MHC-I and II antigens and activating cytotoxic lymphocytes^34^.

Recent data suggested that the overall burden of SCNAs correlated with tumor immune evasion^36^. Also, we observed that the expression of the MHC class I antigen presenting machinery (APM) and angiogenesis signature genes as well as the infiltration levels of T cells or cytotoxic cells were the lowest in cluster C, which had the highest burden of arm-level SCNAs but similar rates of somatic mutations comparing with clusters A and B. This observation suggested that the high burden of arm-level SCNAs may contribute to the poor clinical outcome of patients in cluster C by inhibiting the activity of cytotoxic lymphocytes within tumor microenvironment.

## Discussion

Our current knowledge about the evolution history of ccRCCs was mainly based on findings from multi-region sequencing of a limited number of patients. Most ccRCC patients have startling intra- and inter-tumor heterogeneity and it is necessary to analyze the evolution patterns of ccRCCs in large numbers of patients from different populations. Patients from different areas have different genetic background and expose to different carcinogens during their lifetime. In our study, we identified some population-specific mutational signatures and SCNAs. The mutational signature caused by exposure to aristolochic acid which may be contained in some Chinese herbs was notable in Chinese patients^37^. The mutational signatures (processes 1 and 3) with unknown causes were enriched with subclonal mutations in Japanese cohort. The prevalence of these two novel signatures in population was also quite low and we cannot exclude the possibilities that whether they were linked to different experiments conditions. Future studies with even larger sample sizes are needed to investigate their underlying causes and potential consequences on the genesis of the late-stage mutations during ccRCC evolution.

Our study provided important insights into the evolution processes of ccRCC. The CCF of each somatic event was a surrogate quantifying its prevalence of mutation among the heterogeneous tumor cell populations within a tumor mass. With regard to the distribution of CCFs of all somatic events, the most frequently observed arm-level SCNA, del(3p), had the highest median and lowest coefficient of variation. Thus, del(3p) was considered to be the earliest event acquired during ccRCC evolution. However, all the rest of somatic events exhibited substantial inter-patient variations in their CCFs, suggesting the presence of great heterogeneity in the evolution pattern of ccRCC among different patients. We ranked the frequent somatic events according to their medians of CCFs and found that most of the top ranked events, including del(1p), del(6q), amp(7), del(14q), and *VHL* mutations, showed disparities in their evolution patterns and comprised the early departure points during ccRCC evolution (Fig. 3b). Of these top ranked SCNAs, del(14q) was identified as a subclonal event indicating poor survival in a number of patients independently, highlighting the evaluation of the timing of mutation acquisitions during ccRCC evolution in prognosis analysis. Nevertheless, we also acknowledged that the statistical inference of the clonality of solid cancers by deep sequencing of single biopsy would have some limitations which could be overcome by single-cell sequencing of multiple biopsies in large cohorts of patients.

Characterizing the molecular subtypes of ccRCC is critical for its clinical management. Despite of extensive studies on genetic prognostic markers in ccRCC previously, no study determined the prognostic values of genetic markers according to whether they were clonal or subclonal. Our study demonstrated the importance of discrimination of clonal mutations from subclonal in prognosis analysis for ccRCC. The prognostic power of individual genetic markers was usually limited by their low mutation frequencies in ccRCC. To increase prognostic power, we tried to categorize ccRCC patients into different molecular subtypes based on the CCFs of multiple prognostic somatic events jointly. Of the three prognostic genomic subtypes, two (clusters A and C) were characterized by dismal clinical outcomes. The genomic features and evolution patterns of these different subgroups of ccRCC differed from each other. Almost all patients in cluster B harbored *VHL* mutations and few of them had *BAP1* mutations. *VHL* and *BAP1* mutations had been shown to be indicators of good and poor clinical outcomes, respectively^9,11^. The overall burden of arm-level SCNAs (especially clonal SCNAs) was highest in cluster C, followed by cluster A. Several cluster-specific arm-level SCNAs were also identified in clusters A and C. Patients in cluster B were depleted of arm-level SCNAs. The overall burden of arm-level SCNAs had been shown to be associated with shortened survival in some human solid cancers^36^. Our study demonstrated the possibility of genomic subtyping of ccRCC by integrative analysis of all somatic events which showed associations with the clinical outcomes of ccRCC in either clonal or subclonal states.

It became increasingly clear that there was a close link between the genomic architectures of tumors and the components of immune infiltrates within their microenvironment. Although the prognostic clusters of ccRCC were identified based on their genomic features, the tumor microenvironments of different subtypes also differed greatly from each other. Our analysis showed that arm-level SCNAs or aneuploidy occurred quite early during ccRCC evolution. Paradoxically, it had been shown that aneuploidy or high burden of arm-level SCNAs would increase the immunogenicity of tumor cells during the elimination phase of immunoediting^38^. However, the relative balance between the immunosuppressive cells and the immune cells with antitumor effects determined the fates of tumor cells that would either be suffered from immune escape or elimination. Tumor cell populations or subclones lack of tumor-specific antigens or with impaired APM would escape from immune surveillance. For the relatively short-lived ccRCC patients (clusters A and C) with a high burden of arm-level SCNAs, our data showed that their immunosuppressive tumor microenvironment had a low level of infiltrating CD8^+^ T cells and decreased activities of APM but were infiltrated with high levels of immunosuppressive cells such as Tregs and Th2 cells.

## Methods

### Data sources and sample information

The study was approved by the institutional review boards at Ethics Committee of The First Affiliated Hospital of Xi’an Jiaotong University and informed consent was obtained from each participant. Raw whole-exome sequencing data on the Japanese ccRCC patients were downloaded from the European Genome-phenome Archive (EGA) (accession number: EGAS00001000509) and somatic variants (including SNVs and SCNAs) in the TCGA ccRCC samples with whole-exome sequencing data were downloaded from the Genomic Data Commons (GDC) data portal (http://gdc-portal.nci.nih.gov). Tissue samples from 41 Chinese ccRCC patients were snap-frozen in liquid nitrogen or immersed in RNAlater (Qiagen, Germany) and stored at −80 °C upon resection. Then, we performed whole genome sequencing using the Hiseq 2000 platform following the manufacturer’s instructions (Illumina, San Diego, CA). Genomic DNA extracted from the tissue samples was sheared with a Covaris instrument to an average size of 500 bp and pair-ended reads with the length of 90 bp were generated. After removing the adapters, the sequencing reads were aligned to the reference human genome (hg19) using BWA.

### Clonal state classification of SNVs and SCNAs

All somatic SNVs were called out by the Mutect2 software^20^. All somatic SNVs were further filtered with the following parameters: a read depth of at least 10× in the germline and tumor samples, a maximum of two variant supporting reads in the germline, a minimum tumor variant allele frequency of 10% and a maximum germline variant allele frequency of 2%. The copy number data were segmented with the ReCapSeg software to identify the SCNAs, with all three cohorts being processed by the same standard pipeline as described in GATK documentation provided by the Broad Institute (http://gatkforums.broadinstitute.org/categories/recapseg-documentation)^21^.

We used the ABSOLUTE software (v1.2) to calculate the purity, ploidy and absolute allele-specific DNA copy-numbers of each sample^22^. To ensure the accuracy of clonal inference, samples with a low tumor cell purity (below 20%) were excluded from further analysis. SNVs and SCNAs were defined as clonal if the probability of observing CCF ≥ 0.95 was >0.5 or subclonal otherwise. Except for the analysis of mutational signatures, all SNVs identified in the Chinese ccRCC samples were excluded from inference of clonal architectures of frequently mutated genes due to the relative low sequencing depths of whole-genome sequencing which were insufficient for the detection of low-frequency subclonal mutations. If a gene harbored multiple non-silent SNVs in a patient, we excluded SNVs located in this gene from analysis of clonal architectures.

Arm-level SCNAs were more prevalent than focal SCNAs in ccRCCs, we focused on analyzing the clonal state of arm-level SCNAs^3^. Genomic segments were called out as SCNAs with the following steps: (i) estimation of the modal allelic copy number and determination of the genome doubling events; (ii) calculation of the homolog-specific copy ratios; (iii) identification of allele specific SCNAs; (iv) determination of the absolute copy number of each segment and estimation of the CCFs for the SCNAs and SNVs. For each chromosomal arm in each patient, we divided the cumulative length of the clonal segments or subclonal segments (defined by ABSOLUTE) by the length of the corresponding chromosomal arm. A chromosomal arm would be defined as clonal (or subclonal) if the cumulative percentage of clonal (or subclonal) segments was above 50%. The median CCF of these clonal or subclonal segments from each chromosomal arm was defined as the CCF of the arm-level SCNA. To reduce the background noise, we only analyzed the clonal states of genes with a mutation frequency of 3% or greater and arm-level SCNAs with a frequency of 10% or above.

### Mutational signature analysis

We applied the “als” algorithm in NMF analysis to discover the mutational processes in our study^23–25^. To guarantee that the within-process distance for each process was always minimal, we applied the optimal k-means clustering method to select the optimal process numbers (Supplementary Fig. 6)^23–25^. To compare the relationship between the mutational processes discovered in our study and the 30 COSMIC signatures, we used ‘cosine’ similarities and ‘Pearson’ correlation values to evaluate their differences^26^.

### Clonal or subclonal mutation enrichment analysis

We used permutation test to assess whether a specific gene or arm-level event was enriched with clonal or subclonal mutations. To be specific, for cancer genes with 60 non-silent mutations including 40 clonal and 20 subclonal across 500 separate samples, we would randomly sample 60 non-silent mutations from 500 samples and calculated the observed clonal/subclonal ratios. We repeated 10,000 times, a *P* value of clonal enrichment was obtained by dividing the times when the observed clonal/subclonal ratio was greater than the expected ratio (40/20) by 10,000.

### Molecular subtyping and temporal order of mutations

We applied the default parameters in the NMF R package to perform molecular subtyping. A numerical matrix describing the CCFs of genes mutated at 3% or above and SCNAs altered at 10% or above of ccRCC samples (columns) was constructed. Specifically, each entry in the matrix was the CCF of each gene or SCNA in each sample. To choose the optimal subgroups, we tried from two to six different values, and computed their quality measure of the results (Supplementary Fig. 7). We proposed to take the first value of subgroup number “3” for which the cophenetic coefficient started decreasing as the optimal value of subgroups^39^. We applied the method of constructing the potential temporal order of mutation acquisitions during tumor evolution followed by previous studies^13,27,28^.

### Statistical analysis

Two-sided Mann–Whitney and Fisher’s exact tests were performed with the R functions Wilcox.test and Fisher.test to generate the empirical *P*-values, respectively. *P*-values were adjusted for multiple hypothesis tests using the R function p.adjust with the “fdr” option.

### Survival analysis

Chi-square test statistics in Kaplan-Meier curves were computed using log-rank tests. *P* values were also calculated from multivariate Cox proportional-hazards regression models using the R package "survival".

## Supplementary information

Supplementary Information

Description of Additional Supplementary Files

Supplementary Data 1

Supplementary Data 2

Supplementary Data 3

Supplementary Data 4

## Data Availability

Clinical and sample data were collected from the European Genome-phenome Archive (EGA) (accession number: [EGAS00001000509]) and the Genomic Data Commons (GDC) data portal ([http://gdc-portal.nci.nih.gov]). The raw whole genome sequencing data of Chinese ccRCCs have been deposited at the European Genome-phenome Archive (EGA: https://ega-archive.org) which is hosted at the EBI and the CRG, under accession number: study, EGAS00001003447; dataset, EGAD00001004588.
