# Supplementary material for: Clonal architectures predict clinical outcome in clear cell renal cell carcinoma
Source: Nat Commun. 2019 Mar 18;10:1245. doi: 10.1038/s41467-019-09241-7 (PMC6423009; doi:10.1038/s41467-019-09241-7)
Supplement: Supplementary file 1 — Supplementary Information [file 41467_2019_9241_MOESM1_ESM.pdf]

# Clonal architectures predict clinical outcome in clear cell renal cell carcinoma

**Huang *et al.***

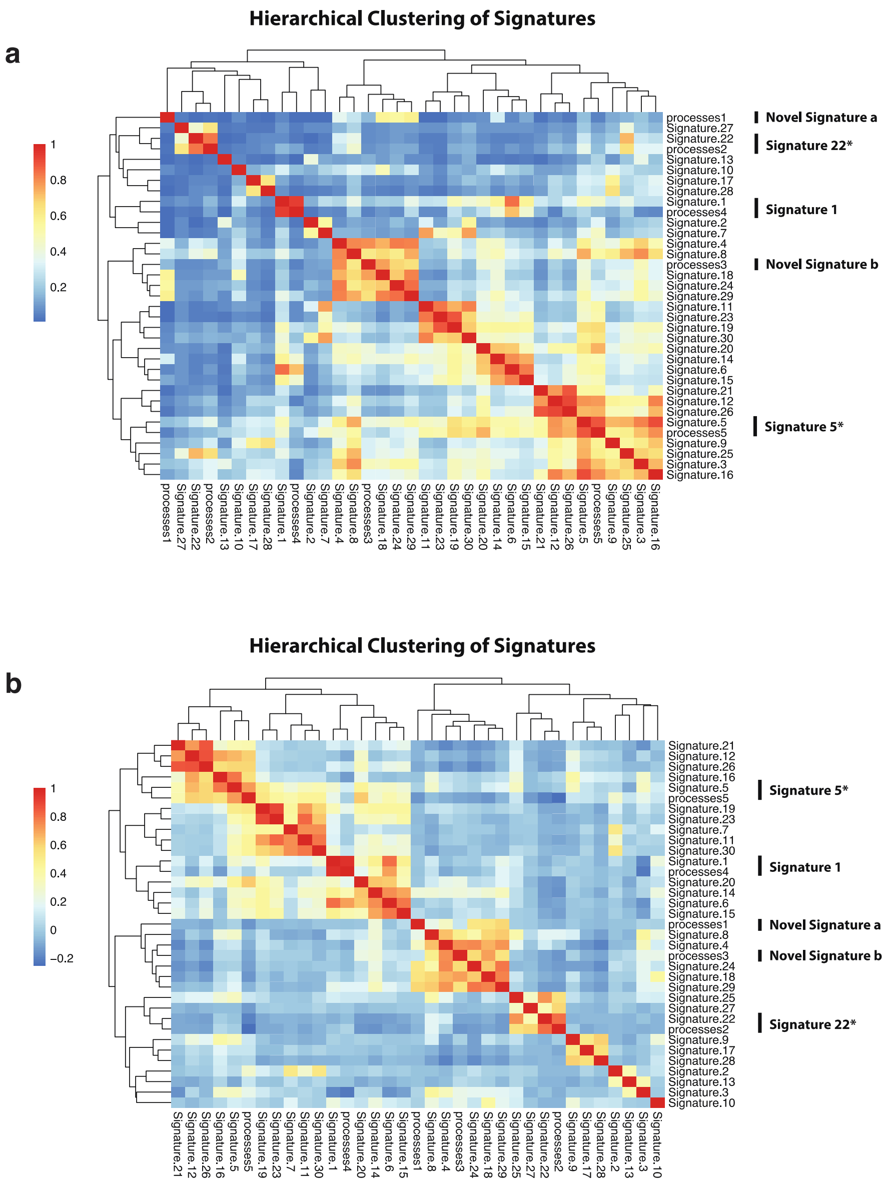


**Supplementary Fig. 1** Unsupervised hierarchical clustering of signatures identified in three ccRCC cohorts (Japanese-104, TCGA-328 and Chinese-41) and the 30 signatures described by COSMIC. **a** Cosine similarity. **b** Pearson correlation. Mutational signature analysis: We applied the “als” algorithm in NMF analysis to discover the mutational processes and applied the k-means clustering method to select the optimal process numbers (Supplementary Fig. 5). We used ‘cosine’ similarities and ‘Pearson’ correlation values to compare the relationship between the mutational processes discovered in our study and COSMIC signatures.

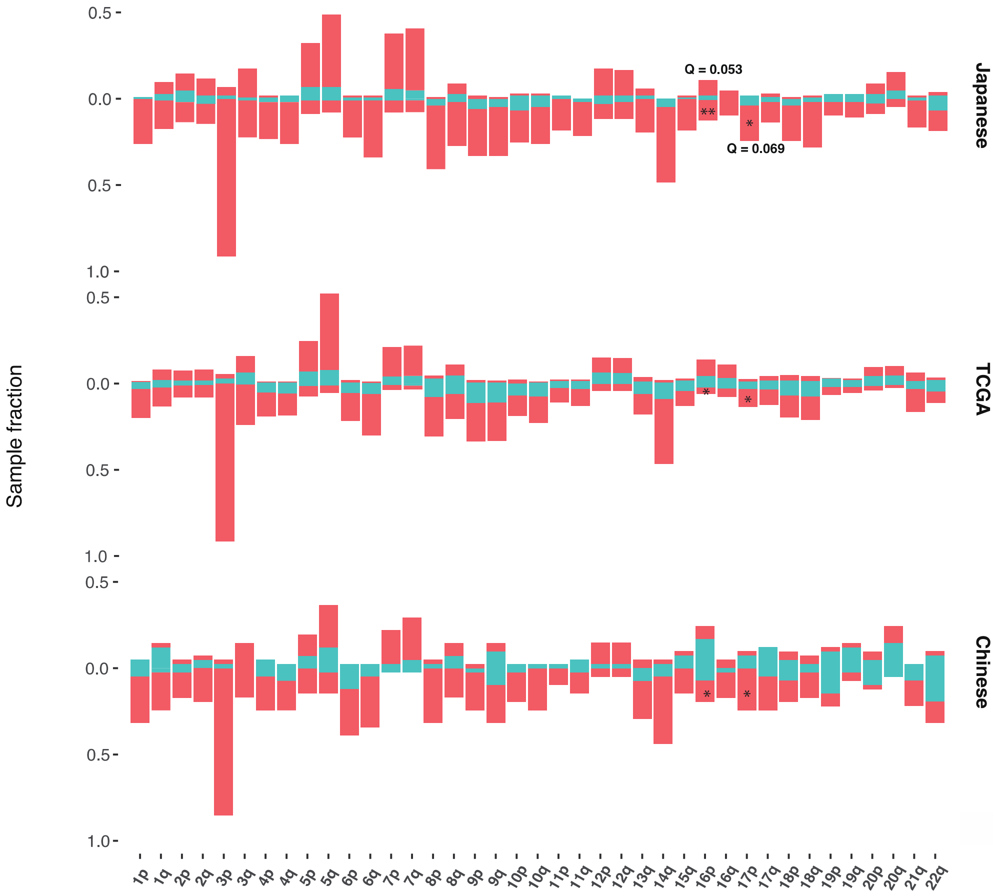


**Supplementary Fig. 2** The clonality of arm-level SCNAs in ccRCC. P values are calculated by fisher test.

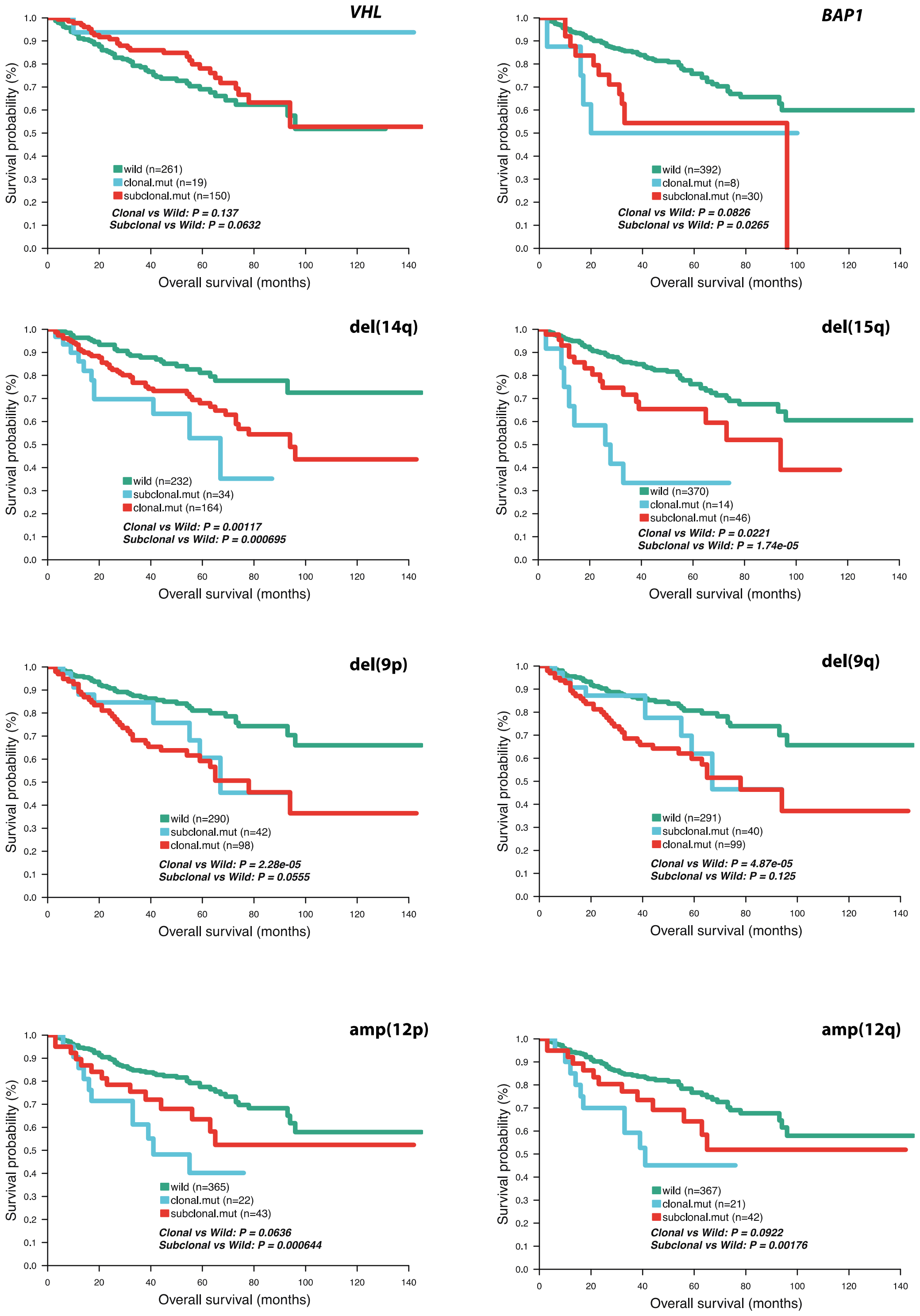


**Supplementary Fig. 3** Prediction of clinical outcome by the clonalities of somatic events. P values are calculated by log Rank sum test.

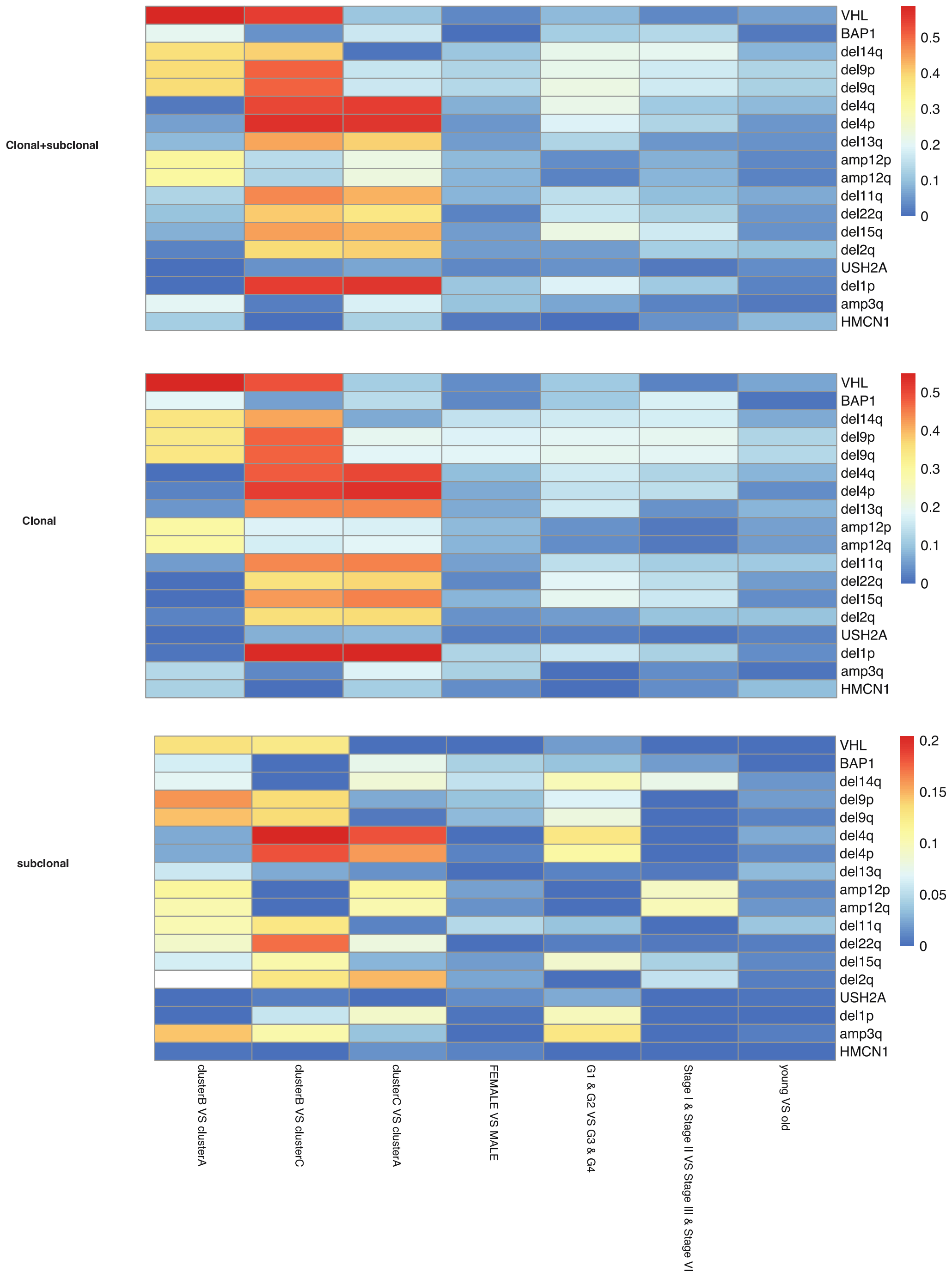


**Supplementary Fig. 4** Association between prognostic events with other clinic factors.

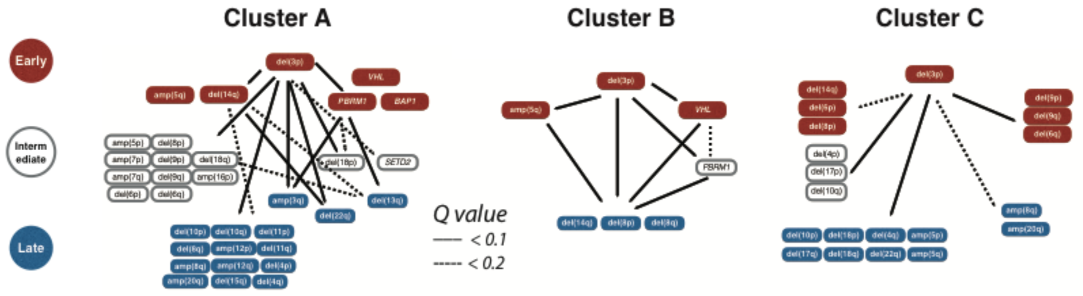


**Supplementary Fig. 5** Inferred evolutionary models of clusters A, B and C.

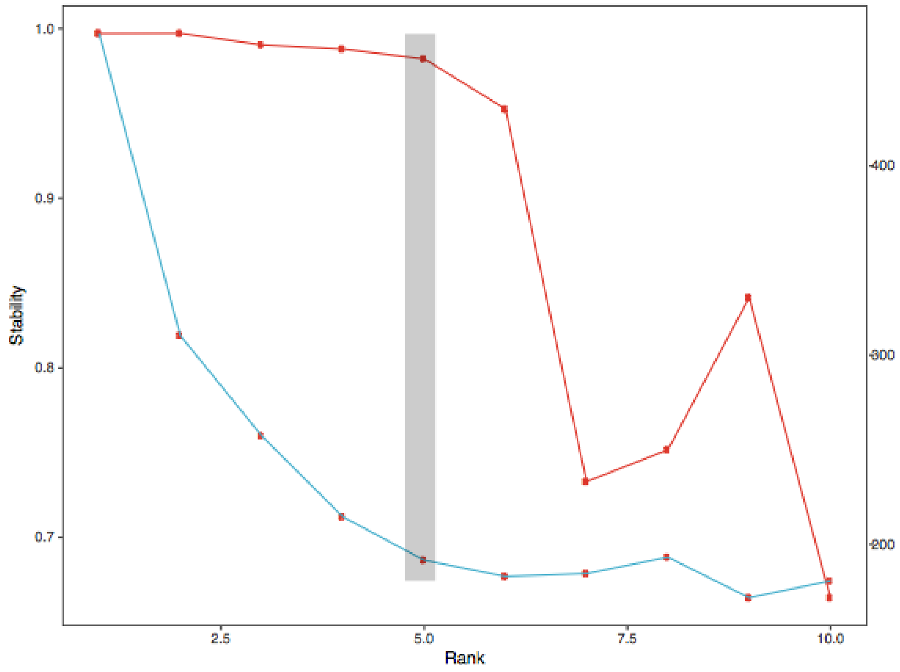


**Supplementary Fig. 6** Stability and Reconstruction Error plot showing the solution for the five mutational signatures.

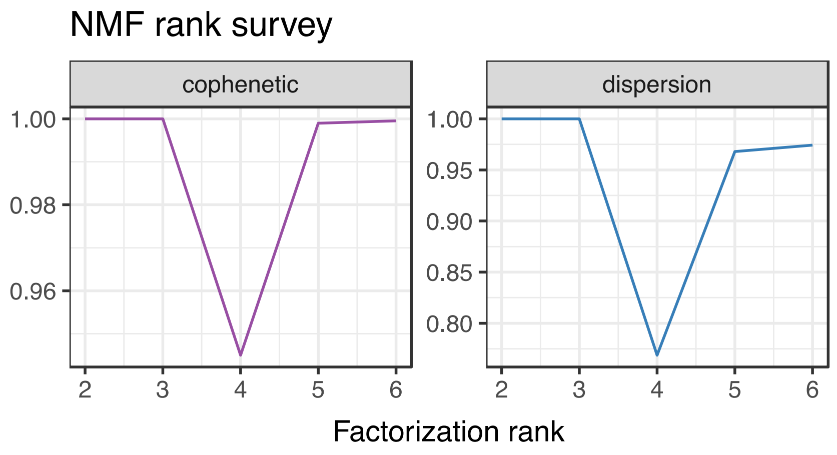


**Supplementary Fig. 7** Estimation of the optimal subgroups: Quality measures computed from 10 runs for each value.
