# Supplementary material for: Clonal architectures predict clinical outcome in clear cell renal cell carcinoma
Source: Nat Commun. 2019 Mar 18;10:1245. doi: 10.1038/s41467-019-09241-7 (PMC6423009; doi:10.1038/s41467-019-09241-7)
Supplement: Supplementary file 2 — Description of Additional Supplementary Files [file 41467_2019_9241_MOESM2_ESM.pdf]

Description of Additional Supplementary Files

File Name: Supplementary Data 1
Description: Comparison of mutational signatures identified in our study to COSMIC mutational signatures.

File Name: Supplementary Data 2
Description: Temporal order of mutation acquisitions in ccRCC patients.

File Name: Supplementary Data 3
Description: Distinct prognostic values of the clonal and subclonal mutations.

File Name: Supplementary Data 4
Description: Predominance of mutation acquisitions in patients with cluster A, B and C ccRCCs.
