# Supplementary material for: Clonal architectures predict clinical outcome in clear cell renal cell carcinoma
Source: Nat Commun. 2019 Mar 18;10:1245. doi: 10.1038/s41467-019-09241-7 (PMC6423009; doi:10.1038/s41467-019-09241-7)
Supplement: Supplementary file 5 — Supplementary Data 3 [file 41467_2019_9241_MOESM5_ESM.doc]

| **Variable** | **Mut vs. Wild** | **Clonal Mut vs. Wild** | **Subclonal Mut vs. Wild** |
| --- | --- | --- | --- |
| ***VHL*** | N = 169 | N = 150 | N = 19 |
|  | HR = 0.626 (0.401 ~ 0.976)  **P = 0.0388** | HR = 0.711 (0.453 ~ 1.11)  *P* = 0.137 | HR = 0.152 (0.0209 ~ 1.11)  *P* = 0.0632 |
|  | HR = 0.514 (0.297 ~ 0.891)  ***P* = 0.0177** | HR = 0.585 (0.338 ~ 1.01)  *P* = 0.0551 | HR = 2.97e−08 (0 ~ Inf)  *P* = 0.995 |
| ***BAP1*** | N = 38 | N = 30 | N = 8 |
|  | HR = 2.42 (1.41 ~ 4.16)  ***P* = 0.00136** | HR =2.28 (1.24 ~ 4.2)  ***P* = 0.00826** | HR = 3.14 (1.14 ~ 8.61)  ***P* = 0.0265** |
|  | HR = 1.79 (0.928 ~ 3.46)  *P* = 0.0824 | HR = 1.66 (0.811 ~ 3.42)  *P* = 0.165 | HR = 2.67 (0.63 ~ 11.3)  *P* = 0.183 |
| ***HMCN1*** | N = 23 | N = 18 | N = 5 |
|  | HR = 1.64(0.792 ~ 3.4)  *P* = 0.182 | HR = 2.17(1.05 ~ 4.49)  ***P* = 0.0369** | HR = 1.1e-07 (NA)  *P* = 0.995 |
|  | HR = 1.85 (0.865 ~ 3.95)  *P* = 0.113 | HR = 2.26 (1.05 ~ 4.88)  *P* = 0.0375 | HR = 2.23e−07 (0 ~ Inf)  *P* = 0.995 |
| ***USH2A*** | N = 14 | N = 12 | N = 2 |
|  | HR = 2.24 (0.82 ~ 6.12)  *P* = 0.116 | HR = 1.75 (0.551 ~ 5.53)  *P* = 0.343 | HR = 15.1 (2.04 ~ 112)  ***P* = 0.00785** |
|  | HR = 2.59 (0.784 ~ 8.54)  *P* = 0.119 | HR = 2.59 (0.784 ~ 8.54)  *P* = 0.119 | NA |

| **Variable** | **Mut vs. Wild** | **Clonal Mut vs. Wild** | **Subclonal Mut vs. Wild** |
| --- | --- | --- | --- |
| **del14q** | N = 198 | N = 164 | N = 34 |
|  | HR = 2.3 (1.47 ~ 3.58)  ***P* = 0.000239** | HR = 2.14 (1.35 ~ 3.39)  ***P* = 0.00117** | HR = 3.35 (1.67 ~ 6.75)  ***P* = 0.000695** |
|  | HR = 1.51 (0.878 ~ 2.58)  *P* = 0.137 | HR = 1.37 (0.782 ~ 2.39)  *P* = 0.272 | HR = 2.78 (1.16 ~ 6.67)  ***P* = 0.0221** |
| **del9q** | N = 139 | N = 99 | N = 40 |
|  | HR = 2.3 (1.51 ~ 3.49)  ***P* = 9.4e-05** | HR = 2.49 (1.6 ~ 3.87)  ***P* = 4.87e-05** | HR = 1.76 (0.855 ~ 3.63)  *P* = 0.125 |
|  | HR = 1.44 (0.839 ~ 2.48)  *P* = 0.185 | HR = 1.48 (0.829 ~ 2.64)  *P* = 0.185 | HR = 1.31 (0.506 ~ 3.41)  *P* = 0.574 |
| **del9p** | N = 140 | N = 98 | N = 42 |
|  | HR = 2.43 (1.6 ~ 3.69)  ***P* = 3.09e-05** | HR = 2.6 (1.67 ~ 4.06)  ***P* = 2.28e-05** | HR = 1.97 (0.984 ~ 3.95)  *P* = 0.0555 |
|  | HR = 1.59 (0.93 ~ 2.73)  *P* = 0.0901 | HR = 1.58 (0.884 ~ 2.82)  P = 0.123 | HR = 1.64 (0.678 ~ 3.98)  P = 0.271 |
| **del1p** | N = 90 | N = 80 | N = 10 |
|  | HR = 1.49 (0.94 ~ 2.37)  *P* = 0.0896 | HR = 1.34 (0.816 ~ 2.19)  *P* = 0.25 | HR = 3.94 (1.42 ~ 10.9)  ***P* = 0.00825** |
|  | HR = 0.838 (0.453 ~ 1.55)  *P* = 0.573 | HR = 0.841 (0.446 ~ 1.59)  *P* = 0.593 | HR = 0.797 (0.107 ~ 5.93)  *P* = 0.825 |
| **del4q** | N = 85 | N = 65 | N = 20 |
|  | HR = 1.62 (1.03 ~ 2.57)  ***P* = 0.0378** | HR = 1.45 (0.87 ~ 2.43)  *P* = 0.153 | HR = 2.36 (1.08 ~ 5.16)  ***P* = 0.0319** |
|  | HR = 0.797 (0.433 ~ 1.47)  *P* = 0.467 | HR = 0.796 (0.416 ~ 1.52)  *P* = 0.491 | HR = 0.804 (0.193 ~ 3.35)  *P* = 0.765 |
| **del4p** | N = 84 | N = 66 | N = 18 |
|  | HR = 1.7 (1.08 ~ 2.69)  ***P* = 0.0222** | HR = 1.61 (0.973 ~ 2.66)  *P* = 0.0637 | HR = 2.11 (0.913 ~ 4.89)  *P* = 0.0805 |
|  | HR = 0.849 (0.462 ~ 1.56)  *P* = 0.599 | HR = 0.922 (0.492 ~ 1.73)  *P* = 0.799 | HR = 0.421 (0.0576 ~ 3.08)  *P* = 0.395 |
| **del13q** | N = 76 | N = 57 | N = 19 |
|  | HR = 1.83 (1.15 ~ 2.93)  ***P* = 0.0113** | HR = 1.86 (1.12 ~ 3.07)  ***P* = 0.0157** | HR = 1.75 (0.632 ~ 4.82)  *P* = 0.283 |
|  | HR = 1.48 (0.827 ~ 2.66)  *P* = 0.186 | HR = 1.45 (0.779 ~ 2.7)  *P* = 0.241 | HR = 1.73 (0.408 ~ 7.31)  *P* = 0.458 |
| **amp3q** | N = 68 | N = 47 | N = 21 |
|  | HR = 1.56 (0.951 ~ 2.58)  *P* = 0.0783 | HR = 1.34 (0.739 ~ 2.42)  *P* = 0.337 | HR = 2.3 (1.06 ~ 5.02)  ***P* = 0.0361** |
|  | HR = 2.02 (1.11 ~ 3.68)  ***P* = 0.0211** | HR = 2.02 (1.06 ~ 3.88)  ***P* = 0.0338** | HR = 2.01 (0.61 ~ 6.64)  *P* = 0.251 |
| **amp12p** | N = 65 | N = 43 | N = 22 |
|  | HR = 2.13 (1.34 ~ 3.38)  ***P* = 0.00136** | HR = 1.73 (0.969 ~ 3.09)  *P* = 0.0636 | HR = 3.07 (1.61 ~ 5.85)  ***P* = 0.000644** |
|  | HR = 1.89 (1.07 ~ 3.34)  ***P* = 0.0293** | HR = 1.34 (0.626 ~ 2.88)  *P* = 0.45 | HR = 2.97 (1.42 ~ 6.2)  ***P* = 0.00379** |
| **amp12q** | N = 63 | N = 42 | N = 21 |
|  | HR = 2.04 (1.27 ~ 3.28)  ***P* = 0.00326** | HR = 1.67 (0.92 ~ 3.02)  *P* = 0.0922 | HR = 2.91 (1.49 ~ 5.68)  ***P* = 0.00176** |
|  | HR = 1.86 (1.04 ~ 3.32)  ***P* = 0.0363** | HR = 1.39 (0.65 ~ 2.96)  *P* = 0.398 | HR = 2.85 (1.31 ~ 6.21)  ***P* = 0.00819** |
| **del11q** | N = 63 | N = 51 | N = 12 |
|  | HR = 1.73 (1.06 ~ 2.83)  ***P* = 0.0278** | HR = 1.6 (0.925 ~ 2.75)  *P* = 0.0929 | HR = 2.37 (0.953 ~ 5.88)  *P* = 0.0634 |
|  | HR = 1.86 (1.04 ~ 3.32)  ***P* = 0.0363** | HR = 0.998 (0.498 ~ 2)  *P* = 0.995 | HR = 1.51 (0.445 ~ 5.16)  *P* = 0.507 |
| **del22q** | N = 54 | N = 33 | N = 21 |
|  | HR = 2.39 (1.47 ~ 3.87)  ***P* = 0.000415** | HR = 2.99 (1.73 ~ 5.17)  ***P* = 8.88e-05** | HR = 1.56 (0.674 ~ 3.59)  *P* = 0.3 |
|  | HR = 1.38 (0.735 ~ 2.59)  *P* = 0.317 | HR = 1.88 (0.937 ~ 3.79)  *P* = 0.0753 | HR = 0.724 (0.222 ~ 2.36)  *P* = 0.593 |
| **del15q** | N = 60 | N = 46 | N = 14 |
|  | HR = 2.39 (1.5 ~ 3.82)  ***P* = 0.00026** | HR = 1.9 (1.1 ~ 3.28)  ***P* = 0.0221** | HR = 5.05 (2.41 ~ 10.6)  ***P* = 1.74e-05** |
|  | HR = 1.44 (0.778 ~ 2.68)  *P* = 0.245 | HR = 1.13 (0.55 ~ 2.31)  *P* = 0.745 | HR = 3.24 (1.25 ~ 8.42)  ***P* = 0.016** |
| **del2q** | N = 41 | N = 35 | N = 6 |
|  | HR = 1.91 (1.08 ~ 3.39)  ***P* =0.0272** | HR = 1.83 (0.994 ~ 3.38)  *P* = 0.0524 | HR = 2.59 (0.633 ~ 10.6)  *P* = 0.185 |
|  | HR = 0.989 (0.475 ~ 2.06)  *P* = 0.976 | HR = 1.03 (0.475 ~ 2.22)  *P* = 0.946 | HR = 0.76 (0.102 ~ 5.68)  *P* = 0.789 |
